# *UGT1A1* genotypes and unconjugated hyperbilirubinemia phenotypes in post-neonatal Chinese children: A retrospective analysis and quantitative correlation

**DOI:** 10.1097/MD.0000000000013576

**Published:** 2018-12-10

**Authors:** Kuerbanjiang Abuduxikuer, Ling-Juan Fang, Li-Ting Li, Jing-Yu Gong, Jian-She Wang

**Affiliations:** a Department of Hepatology, Children's Hospital of Fudan University; b Department of Pediatrics, Jinshan Hospital of Fudan University, Shanghai, China.

**Keywords:** children, Crigler-Najjar syndrome, Gilbert syndrome, bilirubin UDP-glucuronosyltransferase gene, unconjugated hyperbilirubinemia

## Abstract

To retrospectively analyze and quantitatively correlate *UGT1A1* (bilirubin UDP- glucuronosyltransferase gene) genotypes and unconjugated hyperbilirubinemia (UCH) phenotypes among Chinese children.

We retrospectively reviewed UCH patients, quantitatively analyzed genotype–phenotype correlation by comparing with healthy controls. Pfam database, SWISS-model, and Pymol were used for UGT1A1 protein domain analysis and protein modeling for assessing the effect of novel missense variants on protein structure.

Seventy four cases, including 21 prolonged unconjugated hyperbilirubinemia (PUCH), 30 Gilbert syndrome (GS), 22 Crigler-Najjar syndrome type II (CNS-II), and 1 Crigler-Najjar syndrome type I (CNS-I) phenotypes were analyzed. Total of 21 variants, including 7 novel variants (c.764T>A/p.L255Q, c.1112C>T/p.T371I, c.1028C>A/p.S343X, c.1047delG/p.I350YfsX16, c.996 + 5G>C/g.6923G>C, c.287G>A/p.G96E, and c.1142G>A/p.S381N) were found. In the multiple regression model, heterozygous A(TA)7TAA, G71R/P364L, and Y486D/other mutations were significantly associated with increased risk of GS, PUCH, and CNS-II, respectively. Total allele number is significantly associated with GS and CNS-II, with each increase in total allele number, the odds ratio (OR) of having GS and CNS-II increased by 1.46 and 4.47 fold, respectively. Having only functional polymorphisms in *UGT1A1* gene is associated with increased risk of PUCH, and GS with OR values of 5.67 (95% CI: 1.52–21.13), and 3.88 (95% CI: 1.02–14.78), respectively. Having only mutation is associated with significantly increased risk of having GS phenotype (OR: 34.00, 95% CI: 4.65–248.37), but not CNS-II. Polymorphism plus mutation had the strongest association with CNS-II with OR value of 64.80 (95% CI: 7.68–546.41), followed by GS (OR: 4.53, 95% CI: 1.08–19.08).

We detected 7 novel variants, and quantitatively calculated risks of having specific phenotypes using genetic data. Among Chinese children, G71R and P364L is independently associated with PUCH, A(TA)7TAA is associated with GS, and Y486D or other disease-causing mutations were associated with CNS-II. Multiple alleles were associated with more severe phenotypes. Combined variant of G71R+Y486D is a common occurrence among Chinese children with UCH.

## Abstract 2

Supplemental Digital Content is available in the text

## 1 Introduction

Uridine-diphosphoglucuronosyltransferase 1 family, polypeptide A1 (UGT1A1) is the key enzyme that catalyzes the glucuronidation of bilirubin. UGT1A1 protein is mainly expressed in the liver and located in the membrane of smooth endoplasmic reticulum.^[1,2]^ Variations in the bilirubin UDP-glucuronosyltransferase (*UGT1A1*) gene can induce quantitatively different degrees of reduction in UGT1A1 enzyme activity, resulting in an inherited non-hemolytic unconjugated hyperbilirubinemia (UCH). Classification of the Crigler-Najjar syndrome type I (CNS-I, OMIM#218800), Crigler-Najjar syndrome type II (CNS-II, OMIM#606785), and Gilbert syndrome (GS, OMIM#143500) subtypes are largely based on serum bilirubin levels, but believed to be the continuous presentation of a single disorder. Some neonatal hyperbilirubinemia and breast milk jaundice patients may belong to transient familial neonatal hyperbilirubinemia (OMIM#237900), which is related to *UGT1A1* polymorphism or mutation. Although previously believed to be autosomal dominant due to higher frequencies of GS, molecular studies have clearly indicated that a single normal *UGT1A1* allele is sufficient to maintain a normal plasma bilirubin, and almost all cases of UGT1A1 deficiencies transmitted in an autosomal recessive manner which requires homozygous or compound heterozygous alleles.^[3,4]^

Due to possible result of natural selection, frequencies of *UGT1A1* gene variants vary among people with different populations.^[5,6]^ A(TA)7TAA (c.-40_-39dupTA or, c.-40_-39insTA) polymorphism in the promoter region was related to GS among Caucasians,^[7,8]^ while G71R polymorphism in the coding region was related to GS among East Asians.^[9,10]^ With regard to neonatal hyperbilirubinemia, CNS-I, CNS-II phenotypes, frequencies of genetic mutations were also different between Caucasians and East Asians.^[7–18]^

Previous reports on *UGT1A1* sequencing among Mainland Chinese children with hyperbilirubinemia focused on either new-born,^[12,17]^ or small number of CNS cases.^[16,18]^ Full spectrum of genotype–phenotype correlation data on post-noenatal children were lacking. Earlier studies on genotype–phenotype correlation around the world^[7–18]^ largely focused on simple observations and chi square statistics. However, with increasing number of rare cases with genetic testing, more robust statistical methods should be applied to further elucidate the predictive role of genetic variants on disease phenotypes. This is especially true when studying the effects multiple variants that commonly occur in a single patient, because multiple regression analyses that controls for other variants may have a stronger predictive value of a single variant for disease phenotypes. We retrospectively analyzed clinical and *UGT1A1* gene sequencing data of post-neonatal UCH cases clinically diagnosed as having prolonged unconjugated hyperbilirubinemia (PUCH), GS, CNS-II, and CNS-I. Besides using traditional variant frequencies and chi square analyses when comparing cases and healthy controls, we attempted a logistic regression models in order to quantitatively evaluate the independent role of each *UGT1A1* variant, genotype, and total allele number on a specific clinical phenotype. Aims of this study were to quantitatively analyze the *UGT1A1* genotype–phenotype correlation among Chinese children with unconjugated hyperbilirubinemia, to elucidate the clinical significance of complex *UGT1A1* genotypes that occurred in a single patient (multiple SNPs, SNP plus mutation, and multiple mutations), and to expand *UGT1A1* variant spectrum by discovering novel mutations or variants.

## 2 Materials and methods

### 2.1 Ethics

The study protocol conforms to ethical guidelines of the Declaration of Helsinki in 2000, and ethical approval is not required due to it's retrospective nature.

### 2.2 Inclusion/exclusion criteria

Medical records of post-neonatal children with isolated unconjugated hyperbilirubinemia treated on outpatient or in-patient basis in the Liver Center, Children's Hospital of Fudan University, and the Pediatric Department in Jinshan Hospital of Fudan University from January 2007 to December 2014 were retrospectively analyzed. Isolated unconjugated hyperbilirubinemia was defined as total serum bilirubin level greater than 1 mg/dL (17.1 μmol/L) with the proportion of unconjugated bilirubin >80%. Included cases must have clinical, biochemical data, and *UGT1A1* gene sequencing results. Each of these patients had data on physical examination, abdominal ultrasound, liver function test, thyroid function test, and complete blood count with reticulocyte percentage. The overall growth and development status of patients were evaluated during clinic visits. Patients were excluded if they had present or past history of hepatic/hematological diseases, or if there is evidence of hemolysis, infection, liver dysfunction, growth/developmental disorders, or hypothyroidism. In addition, the following exclusion criteria were used: cases with obvious congenital malformations or abnormal growth and development; infants with a history of severe diseases in neonatal period, such as birth asphyxia, parenteral nutrition; and children with preterm birth (<37 weeks’ of gestational age) and/or low birth weight (<2500 g).

Fifty healthy children who were born to hepatitis B antigen positive mother and receiving yearly follow-up were used as healthy controls. Those children have medical record since birth, and none of them had previous history of pathogenic jaundice, positive hepatitis B surface antigen, or any other illnesses. Liver function tests were within normal range during the follow up period.

### 2.3 Genetic testing

One milliliter of venous blood was obtained from each participant, and the whole genome was extracted from peripheral white blood cells using routine methodology. Promoter region, all 5 coding exons, and adjacent regions of *UGT1A1* gene were amplified before Sanger sequencing. List of primers and Sanger sequencing results of novel variants are provided as supplementary materials. Purified polymerase chain reaction (PCR) products were sequenced by laser-induced fluorescence method on ABI Prism 3730 Genetic Analyzer (Applied Biosystems, Foster City, CA). Sequencing data analyses were performed by using BIOEDIT software (North Carolina State University, Raleigh, NC), and double checked by 2 investigators. Genomic sequences obtained at the National Center for Biotechnology Information (NCBI), and sequence RefSeq NG_009254.1 was used as the *UGT1A1* gene sequence reference. Possible genetic variants were confirmed by direct sequencing from both directions using a second independent PCR fragment.

### 2.4 Protein modeling

We analyzed UGT1A1 protein domain structure using Pfam database (http://pfam.xfam.org/protein/p22309), and used similar protein template with UDPGT domain (5gl5.1.A) for constructing UGT1A1 protein model. Protein models for wild-type UGT1A1 protein as well as proteins carrying each of the missense variant (including G96E, L255Q, T371I, and S281N) were constructed with SWISS-model (https://www.swissmodel.expasy.org). Generated protein structures were analyzed with Pymol.

### 2.5 Clinical phenotyping

Criteria for clinical diagnoses for CNS-I, CNS-II, GS, and PUCH phenotypes were adapted from Fabris et al^[19]^ with some modification. CNS-I was diagnosed if the child had serum bilirubin concentration above 25 mg/dL (427.5 μmol/L), and the concentration did not decrease significantly after phenobarbital administration. Patients with CNS-II had serum bilirubin level in the range of 5 to 25 mg/dL (85.5–427.5 μmol/L), and responded to phenobarbital administration. GS, the mildest form, clinically manifested as mild or intermittent unconjugated hyperbilirubinemia and serum bilirubin level fluctuated from normal to 5 mg/dL (85.5 μmol/L). PUCH was defined as post-neonatal cases with serum total bilirubin level higher than 17.1 μmol/L with the proportion of unconjugated bilirubin greater than 80%, and serum bilirubin level normalized within 5 months of age regardless of feeding pattern (breast-feeding or formula feeding).

### 2.6 Statistical analysis

Statistical analyses were performed with STATA software (version 12 Special Edition, STATA Corp, College Station, TX) by the first author (KA) with a qualification of biostatistics. Pearson chi-square test was used to evaluate genotype–phenotype correlations, and Fisher exact values were calculated when expected frequencies might be ≤5. A 2-sided *P*-value of <0.05 was considered statistically significant. Single and multiple logistic regression models were constructed to determine odds ratios (OR) and 95% confidence intervals (95% CI) of having disease phenotypes with all *UGT1A1* variants, allele frequencies, and genotypes.

## 3 Results

### 3.1 Case profiles

**Figure 1:**
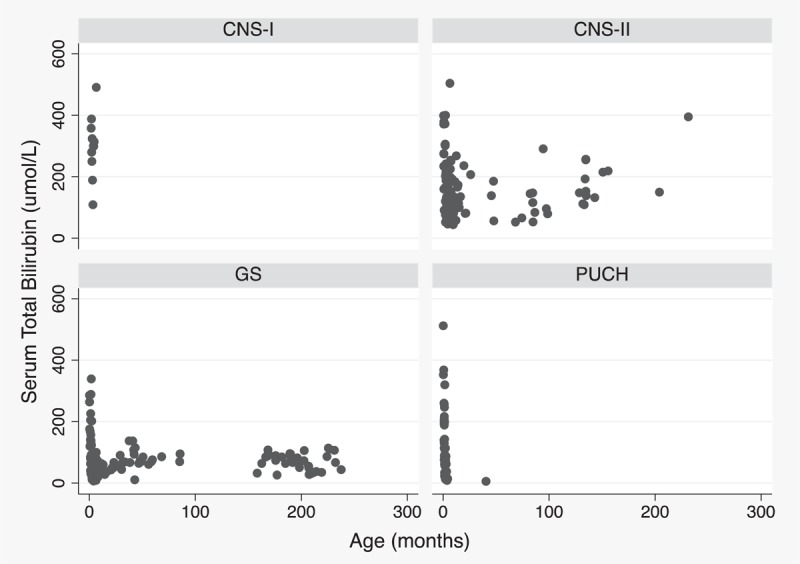
Scatter plot of 304 observations of serum total bilirubin level from 74 patients with CNS-I (1 case), CNS-II (22 cases), GS (30 cases), and PUCH (21 cases). CNS-I = Crigler–Najjar syndrome type I, CNS-II = Crigler–Najjar syndrome type II, GS = Gilbert syndrome, PUCH = prolonged unconjugated hyperbilirubinemia.

Search of in-patient/out-patient records, and genetic test reports from January 2007 to December 2014 revealed 93 post-neonatal patients with unconjugated hyperbilirubinemia and *UGT1A1* gene sequencing record. We excluded 16 patients with gene sequencing data for lack of sufficient clinical information. Another 3 children were not included due to existence of other liver function test abnormalities. Finally, a total of 74 post-neonatal patients with unconjugated hyperbilirubinemia including 21 cases with PUCH, 30 children with GS, 22 patients with CNS-II, and 1 infant with CNS-I, were included in the final analysis. Figure 1 summarized 304 observations of serum total bilirubin levels from 74 patients, and changes according to age by various disease types.

### 3.2 *UGT1A1* gene sequencing results and novel variants

Total of 21 *UGT1A1* variants were found on UCH cases, including 3 variants in the promoter or non-coding region {A(TA)7TAA, c.-64G>C, and c.996 + 5G>C}, 12 missense variants (c.211G>A/p.G71R, c.287G>A/p.G96E, c.625C>T/p.R209W, c.686C>A/p.P229Q, c.764T>A/p.L255Q, c.1007G>A/p.R336Q, c.1091C>T/p.P364L, c.1112C>T/p.T371I, c.1142C>T/p.S381N, c.1352C>T/p.P451L, c.1456T>G/p.Y486D, and c.1471G>A/p.V491I), 4 non-sense/frame-shift variants (c.1021C>T/p.R341X, c.1028C>A/p.S343X, c.1047delG/p.I350YfsX16, and c.1069C>T/p.Q357X), and 2 synonymous variants (c.189C>T/p.63D>D, and c.420G>A/p.L140L).

**Figure 2:**
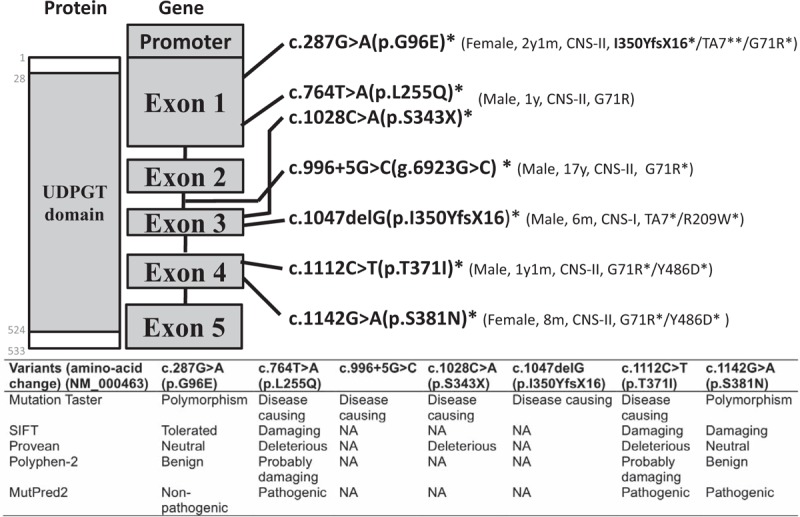
Novel mutations (cDNA position, position on protein sequence with UDP-glucuronosyl transferases (UDPGT) domain, in-silico pathogenicity prediction, patient characteristics, and co-occurrence of other variants. ^∗^: heterozygous mutation; ^∗∗^: homozygous mutation); identified in this study. (RefSeq NG_009254.1, and NM_000463 were used as the *UGT1A1* gene and protein sequence reference.).

**Figure 3:**
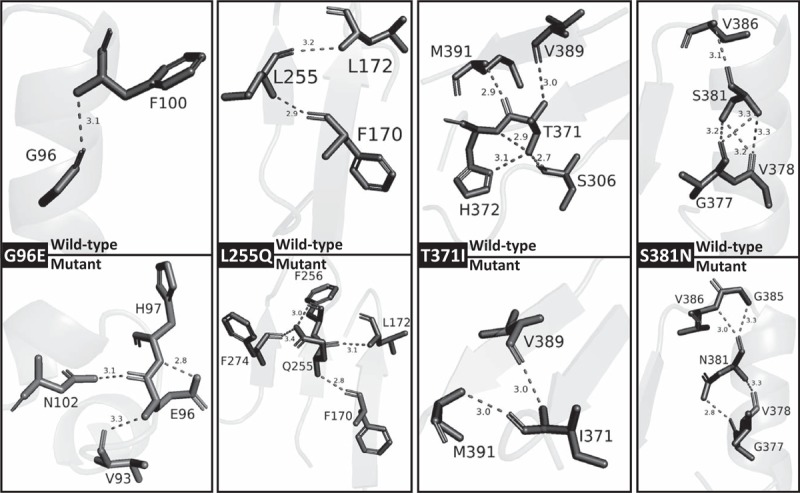
Protein modeling with SWISS-Model: PDB 5gl5.1. A model was used as a template to construct protein models for wild-type UGT1A1 protein as well as proteins carrying each of the missense variant (including G96E, L255Q, T371I, and S281N). Polar contacts with other residues within the protein were altered in all missense variants, and alpha-helix structure surrounding the G96E variant residue was disrupted. UGT1A1 = uridine-diphosphoglucuronosyltransferase 1 family, polypeptide A.

Variants found in our patients were considered novel after comparing with the *UGT1A1* and common exons allele database (http://www.pharmacogenomics.pha.ulaval.ca/cms/site/pharmacogenomics/ugt_alleles), Exome Variant Server (http://evs.gs.washington.edu/EVS/), latest update on *UGT1A1* gene mutation database in 2013,^[15]^ ClinVar records, 1000Genome database (including Han Chinese genome sequenecing data), dbSNP, and Pubmed search. Seven novel *UGT1A1* gene variants (c.287G>A/p.G96E, c.764T>A/p.L255Q, c.996 + 5G>C/g.6923G>C, c.1028C>A/p.S343X, c.1047delG/p.I350YfsX16, c.1112C>T/p.T371I, and c.1142G>A/p.S381N), patient information, other co-occurring variants were described in Fig. 2. All novel protein coding variants were located within the predicted UDPGT domain of UGT1A1 protein. We also included pathogenicity prediction results including MutationTaster (http://www.mutationtaster.org), SIFT (http://provean.jcvi.org), Provean (http://provean.jcvi.org), PolyPhen-2 (http://genetics.bwh.harvard.edu/pph2/), and MutPred2 (http://mutpred.mutdb.org) in Fig. 2. Sanger sequencing results were provided as supplementary file. All novel variants were associated with CNS-I, and CNS-II phenotypes. Polar contacts with other residues within the protein were altered in all novel missense variants, and alpha-helix structure surrounding the G96E variant residue was disrupted (Fig. 3).

### 3.3 UCH cases with triple/more alleles in *UGT1A1* gene

Of 74 patients with UCH, 29 (39%) found to have triple or more alleles in *UGT1A1* gene with 19, 9, and 1 patient had triple, quadruple, and 5 alleles, respectively. Seventeen patients were clinically diagnosed as having CNS-II, and all had at least 2 mutant alleles plus 1 polymorphic allele. Ten patients had GS, 8 had 2 alleles with polymorphisms plus at least 1 mutated allele, while other 2 had one polymorphism and 2 mutations. One patient with PUCH had 3 heterozygous polymorphisms {A(TA)7TAA, G71R, P229Q}. Another patient with CNS-I had heterozygous A(TA)7TAA allele, 2 heterozygous mutations containing 1 non-synony mous mutation (R209W) and 1 single nucleotide deletion resulting a stop codon downstream (c.1047delG/p. I350YfsX16).

Among patients with triple/more alleles, 12 had information related to family screening. Parents of 4 children had genetic testing and origins of variants/mutations were determined. Most screened family members were healthy, except for fathers of 2 children with CNS-II had history of GS, and both parents of 1 child with CNS-II had history of jaundice (Table 1).

**Table 1:**
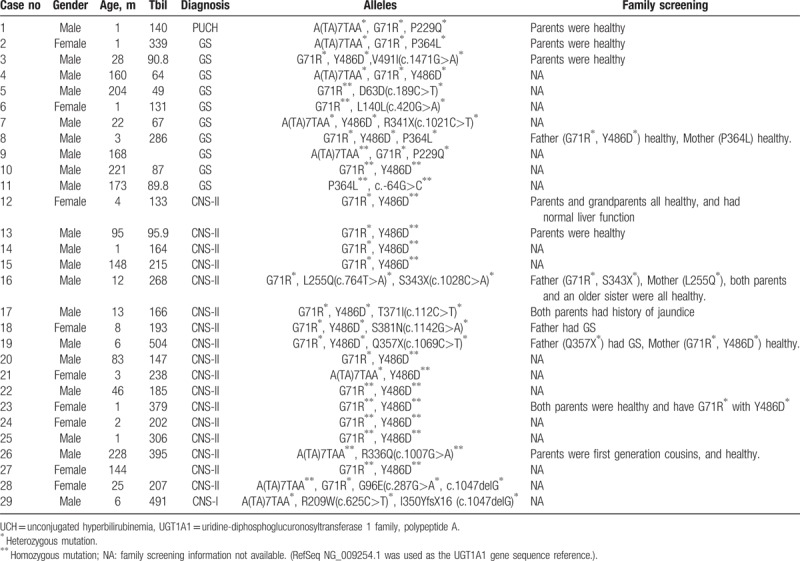
UCH cases with 3 or more alleles in UGT1A1 gene.

### 3.4 UCH cases that cannot be explained by *UGT1A1* gene sequencing

Twenty cases (27%) in our series had normal *UGT1A1* genotype or had only 1 heterozygous allele in *UGT1A1* gene, including 7 children with GS, 2 cases with CNS-II, and 11 infants with PUCH. *UGT1A1* genotype alone cannot explain the rise in serum bilirubin level, because 1 normal allele is sufficient to maintain sufficient enzyme activity. With the exception of 1 GS patient with single Y486D allele and another CNS-II case with single P451L allele, all other patients had normal genotype or one heterozygous polymorphism (G71R or P364L) (Table 2).

**Table 2:**
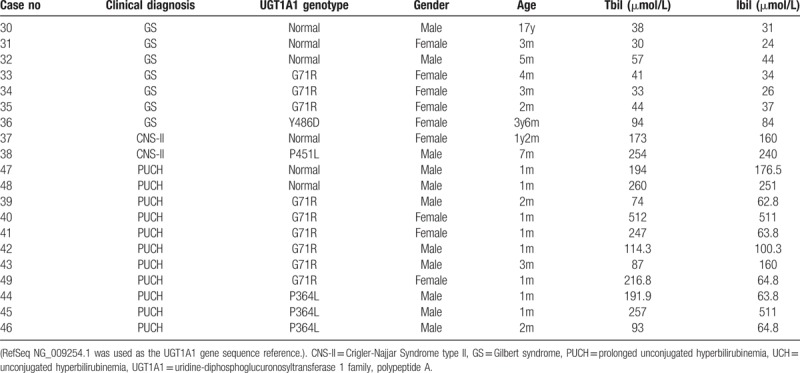
UCH cases unexplained by UGT1A1 genetic analysis.

## 4 Genotype–phenotype correlation

### 4.1 Variant frequencies

We compared frequencies of individual polymorphisms and mutations in healthy controls and different hyperbilirubinemia subtypes. Polymorphisms were more common among patients with hyperbilirubinemia than healthy controls (*P* < .001). Homozygous A(TA)7TAA variant was most commonly occurred in GS patients (12%, *P* = .031). Homozygous G71R is most frequently occurred in CNS-II (27%, *P* = .006), followed by GS (20%) and PUCH (19%). Heterozygous P364L is most prevalent in patients with the PUCH phenotype (29%, *P* = .008). Significantly higher percentage of GS (13%), CNS-II (20%), and CNS-I (100%) patients had genetic mutations compared with healthy controls (0%) and PUCH patients (5%) (*P* < .001). Regardless of homozygosity or heterozygosity, frequencies of Y486D mutation were significantly high among GS and CNS-II cases (*P* = .006, and .000 for heterozygous and homozygous mutation, respectively). For all other mutations, CNS-II cases had the highest percentage of single heterozygous mutation (23%, *P* = .004), while CNS-I infant had the highest proportion of homozygous or double heterozygous mutations (100%, *P* = .001). We also added *P* values (Fisher exact) from chi-square analyses when compared with healthy controls (Table 3).

**Table 3:**
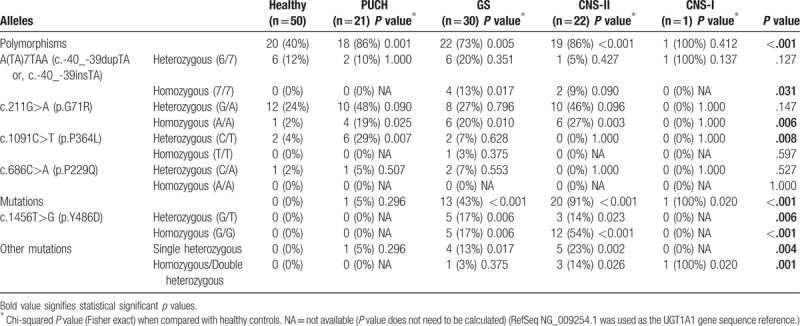
Frequencies of individual polymorphisms, and mutations in healthy controls, and different hyperbilirubinemia subtypes.

### 4.2 Allele characteristics and genotypes

Occurrence of allele types such as promoter/intron, non-synonymous, and insertion/deletion/stop codon are significantly different among various disease phenotypes (*P* = .028, <.001, and .001, respectively). However, there is no significant difference in synonymous allele distribution among different phenotypes (*P* = .131). As for the genotypes of each individual patient, co-occurrence of polymorphism and mutation is significantly different among different phenotypes (*P* < .001), and higher among most severe phenotypes such as CNS II and CNS I. Frequencies of mutation but not polymorphism is highest among patients with GS and CNS-II (*P* = .011). Transient hyperbilirubinemia patients had the most proportion of polymorphisms without mutation (81%, *P* < .001), while normal alleles strongly correlated with healthy individuals (*P* < .001). In terms of allele frequency, phenotype severity increased with the increase of allele number occurred in each individual. Lower number of alleles were more frequent in patients with transient hyperbilirubinemia and GS, while higher number of alleles were associated with CNS-II and CNS-I. Among 74 patients, co-occurrence of G71R and Y486D genotype was encountered in 17 patients, and G71R+Y486D genotype only occurred in GS and CNS-II phenotypes (*P* < .001) (Table 4).

**Table 4:**
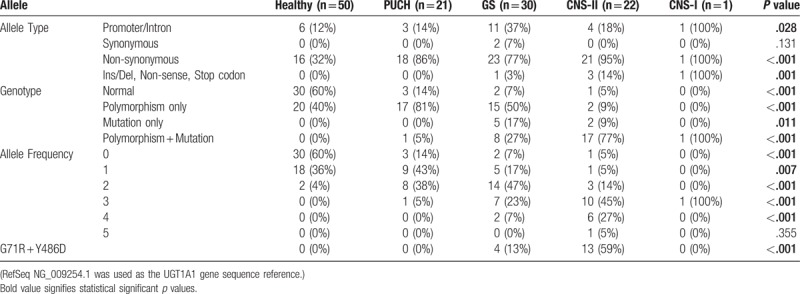
Correlations between clinical diagnosis and allele type, individual genotype, and allele frequency.

### 4.3 Quantitative evaluation of genotype–phenotype correlation

We constructed multiple and single logistic regression models to calculate the risk of having disease phenotypes with individual polymorphisms, total allele numbers, and specific genotypes of each individual. As expected, normal genotypes, which were indicated by constant OR when all genotypes in the model were absent, in all models were significantly associated with decreased odds ratio of having PUCH, GS, and CNS-II phenotypes. CNS-I phenotype was not included in logistic regression model due to limited number of cases (Table 5).

**Table 5:**
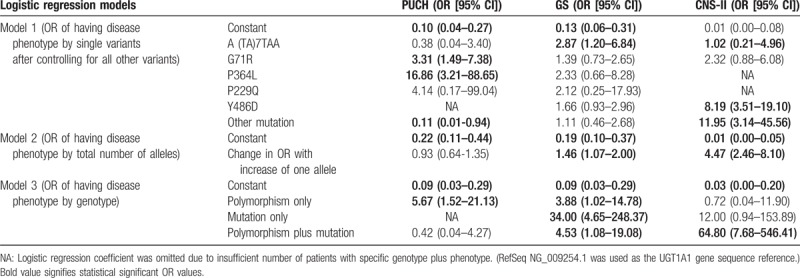
Logistic regression rodels for disease risk calculation by single variants, total allele number, and individual genotypes.

In Model 1 of multiple logistic regression, OR values of having disease phenotypes with 1 unit increase of each specific single allele after adjusting for all other alleles were calculated. The OR of having GS after 1 unit increase in TA7 polymorphism (one unit of TA7 equals to 1 allele) will increase by a factor of 2.83 (95% CI: 1.20–6.84). One unit increase in G71R and P364L alleles were significantly associated with PUCH phenotype with increase of OR values by factors of 3.31 (95% CI: 1.49–7.38) and 16.86 (95% CI: 3.21–88.65), respectively. Presence of single Y486D or other mutated alleles significantly increased the risk of having CNS-II with OR increase by factors of 8.19 (95% CI: 3.51–19.10) and 11.95 (95% CI: 3.14–45.56), respectively. However, presence of mutated alleles other than Y486D is associated with decreased risk of having PUCH phenotype (OR: 0.11, 95% CI: 0.01–0.94). OR value of having disease phenotypes when patients have multiple variants can calculated with this model using the following formula: (OR of TA7)^(number of TA7 allele) ± (OR of G71R)^(number of G71R allele) ± (OR of P364L)^(number of P364L allele) ± (OR of P229Q)^(number of P229Q allele) ± (OR of Y486D)^(number of Y486D allele) ± (OR of Other Mutation)^(number of other mutated alleles). For example, OR of having PUCH in a patient with homozygous G71R and heterozygous P364L is 30.82 (0.38^0 + 3.31^2 + 16.86^1 + 4.14^0 + 0.11^0).

Effect of total allele number in *UGT1A1* gene on disease phenotypes were calculated by single logistic regression in Model 2. Regardless of allele type, each increase of allele is associated with increased OR by factors of 1.46 (95% CI: 1.07–2.00) and 4.47 (95% CI: 2.46–8.10) for having a diagnosis of GS and CNS-II, respectively. In Model 2, OR of having disease phenotype can be calculated with the following formula: (OR value with the increase of one allele)^(total number of allele). For example, OR of having CNS-II when *UGT1A1* gene revealed 3 alleles is 89.31 (4.47^3).

In Model 3 of logistic regression, genotypes of each individual patients (classified as having polymorphism only, mutation only, and polymorphism plus mutation, classicications were mutally exclusive), and OR values of having disease phyenotype were calculated. Having only functional polymorphisms in *UGT1A1* gene is associated with increased risk of PUCH, and GS with OR of 5.67 (95% CI: 1.52–21.13), and 3.88 (95% CI: 1.02–14.78), respectively. Having only mutation is associated with significantly increased risk of having GS phenotype (OR: 34.00, 95% CI: 4.65–248.37), but not CNS-II. Polymorphism plus mutation had the strongest association with CNS-II with OR value of 64.80 (95% CI: 7.68–546.41), followed by GS (OR: 4.53, 95% CI: 1.08–19.08). Since variants in Model 3 is mutually exclusive, OR values of having disease phenotypes by simply adding the constant OR value to the OR values of specific genotye. For example, OR of having GS when *UGT1A1* gene sequencing result showed mutations without polymorphism is 3.88.

## 5 Discussion

This is, to date, the largest series of post-neonatal UCH cases in mainland China describing *UGT1A1* gene sequencing results in 74 cases with well defined disease phenotypes (PUCH, GS, CNS-II, and CNS-I). Genetic tests revealed 15 known variants, and discovered 7 novel variants including 6 possibly disease-causing mutations and 1 polymorphism as predicted by various in-silico pathogenicity prediction tools (Fig. 2).

Twenty-nine of our 74 UCH cases carried triple or more alleles in *UGT1A1* gene. All CNS-I and CNS-II patients had at least 2 mutant alleles plus 1 allele with polymorphism, while the majority of GS subjects had 2 alleles with polymorphisms plus at least 1 mutated allele. One PUCH patient carried 3 heterozygous polymorphisms. Among 12 patients with genetic or clinical information related to family screening, most family members were healthy, except for fathers of 2 children with CNS-II had history of GS, and both parents of 1 child with CNS-II had history of jaundice (Table 1). There are some reports of UCH cases carrying triple/more *UGT1A1* variants. Maruo et al^[20]^ reported a triple homozygous mutation in *UGT1A1* gene [T-3279G, A(TA)7TAA, and H39D] in a CNS-II patient, and co-occurrence of 3 other mutations in a family with CNS-I and GS.^[21]^ Our report and other studies^[20–22]^ further emphasized the importance of family screening among UCH cases with triple/more alleles.

Skierka et al^[23]^ examined 181 *UGT1A1* gene sequencing reports, and identified 34 UCH cases (19%) with no identifiable or only 1 copy of *UGT1A1* variant. UGT1A1 enzyme deficiency cannot be explained by their genotype, since 1 normal allele is sufficient to maintain normal enzyme activity. In our cohort, higher percentage of cases (27%) had insufficient genetic evidence to explain UCH (Table 2). Although majority of cases were screened for G6PD enzyme deficiency, phenobarbital-responsive enhancer module (PBREM) was not covered when *UGT1A1* gene sequencing and genetic testing for genes other than *UGT1A1* were not conducted. Chen et al^[24]^ reported that PBREM and A(TA)7TAA had high linkage disequilibrium among Chinese adults, and functional studies indicated that the combination of PBREM and A(TA)7TAA decreases the expression of *UGT1A1* gene.^[25]^ More detailed *UGT1A1* genetic sequencing including PBREM and large insertion/deletion, and sequencing for other genes that were reported to cause UCH, such as solute carrier organic anion transporter family member 1B1 (SLCO1B1),^[26,27]^ glucose-6-phosphate dehydrogenase (G6PD),^[28]^ and Glutathione S-transferases (GSTs)^[29,30]^ may shed some light into this phenomenon. Future studies should focus on genetically unexplained UCH cases and find plausible disease causing mechanisms.

Frequencies of common functional variants differ significantly among people with different skin colours (African, Caucasian, and East Asian).^[5,6,31,32]^ A(TA)7TAA variant is significantly associated with breast milk jaundice among Europeans,^[33]^ but not among the East Asians.^[34,35]^ In our analysis of 74 children with isolated unconjugated hyperbilirubinemia, frequencies of heterozygous A(TA)7TAA variant among all phenotypes (including healthy controls) were similar, but the frequency of homozygous A(TA)7TAA is highest among patients with GS and significantly increases the risk of having GS phenotype. Similar to other studies from East Asia, G71R, but not A(TA)7TAA, significantly increased the risk of PUCH (which included some patients with breast milk jaundice) in our cohort.

Huang et al^[36]^ first discovered P364L variant in a Taiwanese GS patient, and Takeuchi et al^[37]^ reported P364L variant reduced UGT1A1 enzyme activity to 64.4% when compared with the wild type. Previous study involving adult Chinese population^[24]^ calculated the P364L carrier rate as 1.67%, and was not associated with elevated bilirubin levels. P364L variant was present in 2 Indian cases with UCH,^[38]^ but not found in previous studies involving European^[33]^ and Japanese^[34]^ patients, and exact carrier rate of P364L among other populations were unknown. Frequencies of P364L among healthy controls, PUCH, and GS cases in our cohort were 4, 29, and 10%, and it is strongly increased the risk of having PUCH phenotype after adjusting for all other variants.

On the other hand, P229Q allele {which co-occurred with the A(TA)7TAA variant in all patients} frequencies were similar among all groups, and P229Q does not seem to increase the risk of UCH phenotypes when analyzed with multiple logistic regressions. Aono et al^[9]^ reported 2 GS cases with P229Q, but significance of this variant was not confirmed with healthy control subjects.

In order to quantify the genotype–phenotype correlation, we constructed multiple and single logistic regression models to predict phenotypes from each variant, genotype, and total allele number. Multiple logistic regression analysis revealed that after adjusting to all other variants, A(TA)7TAA was independently associated with GS, both G71R and P364L were significantly associated with PUCH, and Y486D or other mutations were associated with CNS-II. Presence of other mutation is negatively associated with, or protective against PUCH. Total allele number is positively associated with GS and CNS-II, and each unit increase of total allele number increased the risk of GS and CNS-II. Chen et al^[24]^ quantitatively analyzed *UGT1A1* variants from 104 UCH cases and 104 healthy controls. Homozygous G71R and A(TA)7TAA were significantly associated with UCH with OR values of 14.93 and 17.79, respectively. However, P364L was not associated with adult UCH. After adjusting for all other genotypes, having only polymorphisms in *UGT1A1* gene is associated with increased risk of PUCH and GS. Having only mutation is associated with significantly increased risk of having GS, but not CNS-II. Polymorphism plus mutation had the strongest association with CNS-II. Most importantly, risks of having PUCH, GS, or CNS-II can be precisely calculated by using algorithms generated by Model 1, 2, and 3 (Table 5). Our case series indicated that 4 cases of GS and 13 children with CNS-II carried G71R + Y486D combined variant.

Comparison of single chi-squared analyses and multiple logistic regression analyses: only the homozygous A(TA)7TAA, but not heterozygous allele, was associated with GS in chi-squared analysis (Table 3). However, when adjusted for all other variants, both heterozygous and homozygous A(TA)7TAA were associated with GS (Table 5). Homozygous G71R was associated with PUCH, GS, and CNS-I in chi-squared analyses (Table 3). But, both heterozygous and homozygous G71R was associated only with PUCH when adjusted for all other variants. Only the heterozygous P364L allele was associated with PUCH in chi-squared analysis (Table 3), but both heterozygous and homozygous P364L was associated PUCH in the multiple regression model (Table 5). Y486D allele was only associated with CNS-II in the multiple regression analyses (Table 5), and chi-squared association of this allele with GS (Table 3) may be due to cofounding of other alleles. Other mutations were associated with GS in chi-squared analysis but not in multiple regression analysis. Other mutations were protective against PUCH when adjusted for all other alleles (Table 5), when no association was observed in chi-squared analysis.

This study population included Han Chinese from all across China, and results may not be generalized to other ethnic minority population which consists of roughly 8% of total population in the country.

## 6 Conclusion

We described 74 post-neonatal cases with well defined clinical UCH phenotypes, conducted a detailed quantitative correlation to *UGT1A1* genotypes, and reported 7 novel *UGT1A1* variants. Risks of having UCH, GS, and CNS-II disease phenotypes can be quantitatively calculated using genetic data. Among Chinese children, G71R and P364L is independently associated with PUCH, A(TA)7TAA is associated with GS, and Y486D or other disease-causing mutations were associated with CNS-II. Multiple alleles were associated with more severe phenotypes. Combined variant of G71R + Y486D is a common occurrence among Chinese children with UCH.

## Author contributions

Jian-She Wang conceived the study, conducted the diagnosis, treatment, and follow-up of patients with unconjugated hyperbilirubinemia, supervised over the genetic testing, and approved the submission of final manuscript. Kuerbanjiang Abuduxikuer, Ling-Juan Fang collected patient files, conducted genetic testing, performed statistical analyses, and wrote the manuscript. Kuerbanjiang Abuduxikuer conducted protein modeling using SWISS-model and Pymol software. Li-Ting Li collected patient files and conducted genetic testing. Jing-Yu Gong collected patient files and participated in patient management.

**Conceptualization:** Kuerbanjiang Abuduxikuer, Jian-She Wang.

**Data curation:** Kuerbanjiang Abuduxikuer, Ling-Juan Fang, Li-Ting Li, Jing-Yu Gong, Jian-She Wang.

**Formal analysis:** Kuerbanjiang Abuduxikuer, Ling-Juan Fang, Jian-She Wang.

**Investigation:** Kuerbanjiang Abuduxikuer, Ling-Juan Fang, Li-Ting Li, Jing-Yu Gong, Jian-She Wang.

**Methodology:** Kuerbanjiang Abuduxikuer, Ling-Juan Fang, Li-Ting Li, Jian-She Wang.

**Project administration:** Kuerbanjiang Abuduxikuer.

**Resources:** Ling-Juan Fang, Li-Ting Li, Jian-She Wang.

**Software:** Kuerbanjiang Abuduxikuer.

**Supervision:** Jian-She Wang.

**Validation:** Kuerbanjiang Abuduxikuer, Jian-She Wang.

**Visualization:** Kuerbanjiang Abuduxikuer.

**Writing – original draft:** Kuerbanjiang Abuduxikuer, Ling-Juan Fang, Li-Ting Li.

**Writing – review & editing:** Kuerbanjiang Abuduxikuer, Jian-She Wang.

## Supplementary Material

Supplemental Digital Content

## Supplementary Material

Supplemental Digital Content

## Supplementary Material

Supplemental Digital Content

## Supplementary Material

Supplemental Digital Content
