# Supplementary material for: UGT1A1 genotypes and unconjugated hyperbilirubinemia phenotypes in post-neonatal Chinese children: A retrospective analysis and quantitative correlation
Source: Medicine (Baltimore). 2018 Dec 10;97(49):e13576. doi: 10.1097/MD.0000000000013576 (PMC6310575; doi:10.1097/MD.0000000000013576)
Supplement: Supplemental Digital Content [file medi-97-e13576-s001.docx]

| Supplementary table：Primers used for sequencing UGT1A1 gene | | | |
| --- | --- | --- | --- |
| Amplificaton Region | Primer name | 5’-3’ Sequence | PCR Product (bp) |
| Promoter | TA-FP | AGCCAGTTCAACTGTTGTTGC | 672 |
|  | TA-RP | AAGGAAAGGGTCCGTCAGC |  |
| Exon l | Exl-FP | GACACAGTCAAACATTAACTTGGT | 1127 |
|  | Ex1-RP | TGATGCCAAAGACAGACTCAAAC |  |
| Exon 2 | Ex2-FP | GGAAGTAAAGGAGAGGAAAATGC | 483 |
|  | Ex2-FP | AACAATGACAACAACCACAACAA |  |
| Exon 3/4 | Ex3/4-FP | AAGTTGCCAGTCCTCAGAAGC | 851 |
|  | Ex3/4-RP | TTTGAAACAACGCTATTAAATGCT |  |
| Exon 5 | Ex5-FP | AGGCAGGAGGATGGCTTG | 753 |
|  | Ex5-RP | GGGGGCACGATACATATTCA |  |
