# Supplementary figures and images for: UGT1A1 genotypes and unconjugated hyperbilirubinemia phenotypes in post-neonatal Chinese children: A retrospective analysis and quantitative correlation
Source: Medicine (Baltimore). 2018 Dec 10;97(49):e13576. doi: 10.1097/MD.0000000000013576 (PMC6310575; doi:10.1097/MD.0000000000013576)

## Slide 1
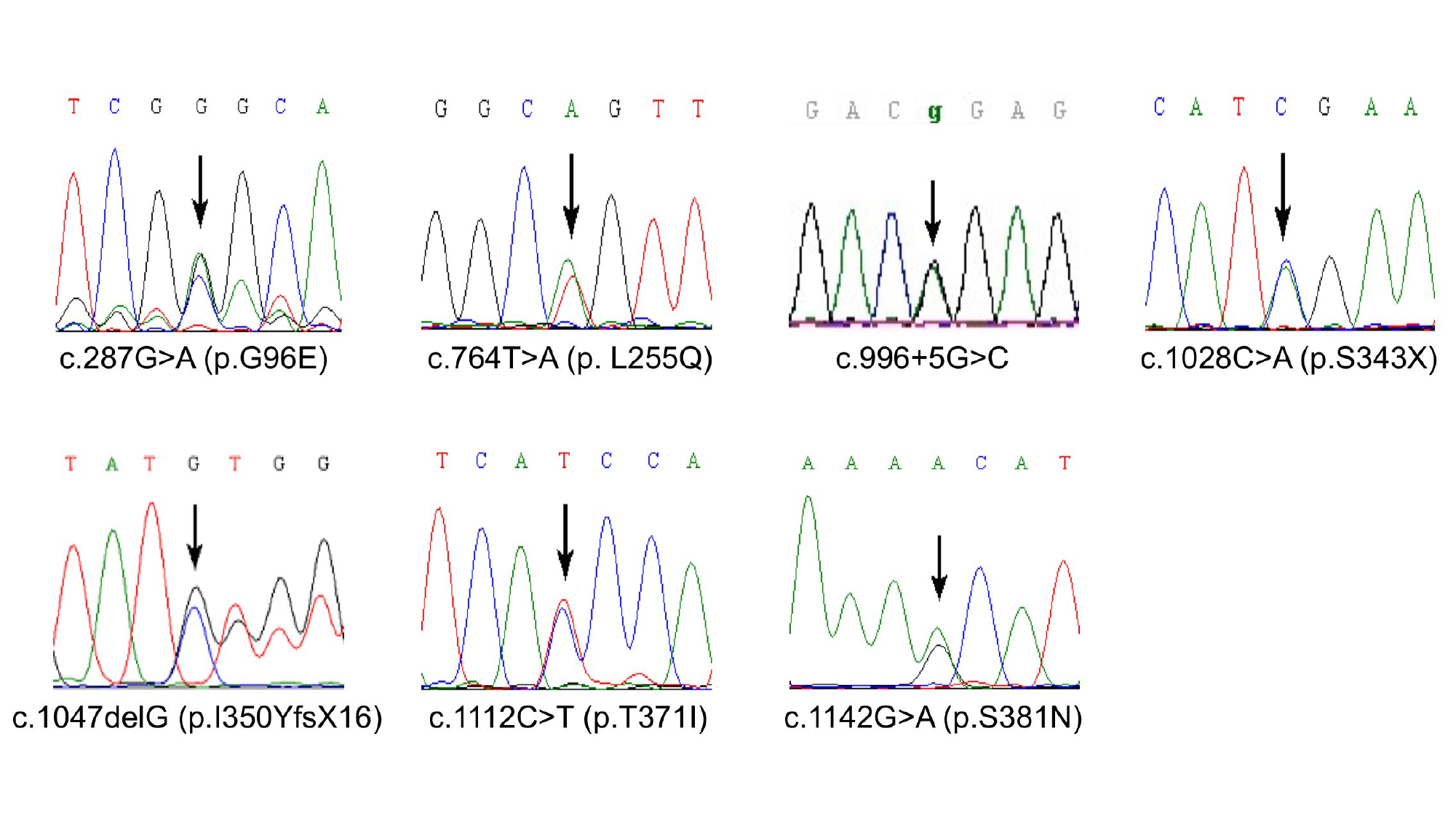

Supplement: Supplemental Digital Content [file medi-97-e13576-s002.pptx]
